# Locus of Control Moderates the Relationship Between Exposure to Bullying Behaviors and Psychological Strain

**DOI:** 10.3389/fpsyg.2019.01323

**Published:** 2019-06-06

**Authors:** Iselin Reknes, Gintare Visockaite, Andreas Liefooghe, Andrey Lovakov, Ståle V. Einarsen

**Affiliations:** ^1^Department of Psychosocial Science, University of Bergen, Bergen, Norway; ^2^Surrey Business School, Department of People and Organisations, University of Surrey, Guildford, United Kingdom; ^3^Department of Organizational Psychology, Birkbeck, University of London, London, United Kingdom; ^4^Center for Institutional Studies, National Research University Higher School of Economics, Moscow, Russia

**Keywords:** workplace bullying, psychological strain, internal locus of control, external locus of control, personal resources

## Abstract

Workplace bullying is regarded as one of the most devastating stressors at work for those targeted, and the bullying-mental health relationship is well-documented in the literature, even under lower levels of exposure. However, less is known about when and for whom these negative behaviors have more effect. Perceived control over outcomes in life (i.e., internal locus of control) has normally been related to good health and well-being, while relying on chance and/or powerful others (i.e., external locus of control) have been related to stress and poor health. In situations with reduced individual control like bullying, however, these mechanisms may act differently. Hence, the aim of the present study was to investigate whether internal and external locus of control, respectively, moderates the bullying-mental health relationship. Data were gathered in 2014–2015 from 1474 Russian employees (44% response rate), and analyzed using Mplus and SEM modeling. Included measurement scales were the Negative Acts Questionnaire-Revised, the General Health Questionnaire-12, and Levenson’s Locus of Control scale. Although the prevalence of high intensity bullying was low, the results showed the expected positive relationship between exposure to bullying behaviors and psychological strain. Furthermore, this relationship was moderated by locus of control. In line with our expectations, internal locus of control did not have the generally assumed positive effect on strain when exposed to bullying behaviors. On the other hand, external locus of control seems relatively beneficial when facing bullying behaviors. The results of this study thus support that exposure to bullying and its associated behaviors are unique stressors where personal characteristics seem to play a different role than normally expected when facing other kinds of stressors.

## Introduction

Over the past 20 years, research has provided converging results from many countries suggesting that exposure to some level of mistreatment and harassment at work is a severe stressor that occurs across all sectors in working life, finding its targets among all age groups, all organizational levels, as well as affecting men and women alike (Zapf et al., 2011; Nielsen and Einarsen, 2018). Such exposure in its more extreme forms, often denoted as workplace bullying, is characterized by three central criteria; repeated exposure to negative and unwanted behavior by other organization members, over a period of time, with a real or perceived imbalance in power between the target and the perpetrator, preventing the target from effectively retaliating in kind (Einarsen et al., 2011). Hence, repetition, intensity, and duration are central parts of the phenomena, often with the victim experiencing lack of resources to stop or neutralize this negative behavior. Furthermore, bullying is not an either or phenomenon, but rather a gradually evolving process where the target is ever more victimized by this systematic mistreatment by peers and superiors. Typical behaviors involved in early phases are often of low intensity, being subtle and indirect forms of psychological aggression targeting either the work situation or personal integrity of the focal person. Later on in the process more open and direct acts occur, where acts of social exclusion, intimidation and even threats of physical aggression may take place. Yet, even in less extreme forms, exposure to such workplace bullying behaviors, by some denoted as incivility (Cortina et al., 2001), is related to most indicators of reduced well-being among its targets. In this, workplace bullying has been related to a range of negative health outcomes, like sleep problems (Niedhammer et al., 2009; Vedaa et al., 2016), burnout (Einarsen et al., 1998; Allen et al., 2015), post-traumatic stress (Nielsen et al., 2015), and mental health problems (Nielsen and Einarsen, 2012; McTernan et al., 2013; Plopa et al., 2016), empirically shown in both cross-sectional and longitudinal studies. Moreover, targets of bullying tend to have higher rates of sickness absence compared to non-exposed employees (Kivimäki et al., 2000; Niedhammer et al., 2009). Most studies on health outcomes of workplace bullying have, however, focused on direct cause-and-effect relationships between variables. Meanwhile, there is a shortage of theory-driven studies suggesting more complex understanding of how and when exposure to bullying influences the health of those targeted (Nielsen et al., 2016; Nielsen and Einarsen, 2018).

Despite the overwhelming evidence indicating stressor–strain relationship between bullying and health, not all targets react in the same way or to the same degree when facing this predicament (Glasø et al., 2007; Rai and Agarwal, 2018). First of all, a work stressor such as exposure to bullying, being perceived as threatening and somewhat out of one’s control, seems to have more severe detrimental effect on targets’ health and well-being than do other comparable stressors (Hauge et al., 2010; Reknes et al., 2017). Secondly, such perceptions and reactions are, however, likely to be influenced by personal dispositions, individual coping strategies, and one’s perceived control over the situation (Spector and O’Connell, 1994; Nielsen and Knardahl, 2015), which is in line with most stress theories. As lack of control is a typical characteristic and outcome of the bullying process (Zapf and Einarsen, 2005), and as studies investigating personal dispositions as moderators in the bullying-strain relationship are called for, the aim of the present study was to investigate whether differences in perceived control over outcomes in life (i.e., locus of control) play a role in the relationship between exposure to bullying behaviors at work and psychological strain (i.e., mental health outcomes). Generally, higher internal locus of control as a personality disposition is related to well-being and good mental health (e.g., Ng et al., 2006). Hence, we expect targets with high internal locus of control to be less affected by bullying. However, in some situations an external locus of control may actually be more adaptive. For instance, in bullying situations where the target in fact has reduced control over the outcome, and experienced series of failed conflict management attempts (Zapf and Einarsen, 2005), we may expect that those with an external locus of control will fare better when facing some level of exposure to bullying (Moreno-Jiménez et al., 2007). People with high internal locus of control may on the other hand experience that their expectancy of being in control is not met. Hence, the present study aims to investigate the possible moderating role of locus of control on the already documented relationship between exposure to bullying behaviors and mental health outcomes, envisioned to suggest significant new practical and theoretical implications.

### Theoretical Background

Locus of control refers to the tendency to perceive outcomes in life as a result of one’s own actions and thus being within one’s own control (i.e., internal locus of control), as opposed to being determined by external factors, such as chance or powerful others (i.e., external locus of control) (Rotter, 1966; Keenan and McBain, 1979). People with high internal locus of control typically try to master their environment, while those with high external locus of control often feel helpless because they perceive that outcomes in life are outside their own control (Keenan and McBain, 1979). Locus of control was initially described as a personality trait referring to a person’s stable beliefs of personal efficacy (Rotter, 1966). Later, however, locus of control has also been described as a coping resource facilitating certain coping styles (Lazarus and Folkman, 1984; Newton and Keenan, 1990; Van den Brande et al., 2016). Illustrative of this, placing the cause of an outcome upon others (i.e., external locus of control) has been related to avoidance coping/ resignation, greater stress and poor health (Evers et al., 2000; Gianakos, 2002; Gore et al., 2016). Internal locus of control, on the other hand, has been associated with help-seeking and positive thinking, as well as lower levels of work stress in general (Gianakos, 2002; Gray-Stanley and Muramatsu, 2011; Gore et al., 2016). Although the moderating role of locus of control in the relationship between workplace bullying and psychological strain has received little attention so far (see Moreno-Jiménez et al., 2007; Rai and Agarwal, 2018), the relationship may be theoretically explained by the framework of the conservation of resources (COR) theory (Hobfoll, 1989). This theory proposes that individuals strive to build and maintain valued resources in their lives, including objects, conditions, energies, and personal characteristics. Hence, stress is regarded as a reaction to situations which threaten with loss of resources, result in an actual loss of resources, or lack of an expected gain in resources is present. According to the theory individual differences act as resources that may affect how individuals react to stress (Hobfoll, 1989), with locus of control being regarded as a particularly important resource in such situations (Newton and Keenan, 1990). More specifically, the level of perceived control in stressful situations is closely related to people’s causal explanations of negative events. According to attribution theory the cause of an outcome may be perceived to either reside within the person (internal orientation) or outside of the person (external orientation) (Heider, 1958). An internal locus of causation is related to seeing negative outcomes in life as caused by personal characteristics like mood, abilities, and personality, while an external locus of causation is related to seeing negative outcomes as caused by situational factors like the nature of the situation, luck, or social pressure (Crisp and Turner, 2007). If the person believes that the cause of one’s treatment by peers or superiors resides within him/her, he or she may be more negatively affected because perceived accountability (see Weiner, 1986). If the person places the cause of the negative behaviors outside him/her, the behavior may be more easily rationalized and the negative outcomes may actually be less severe.

So, theoretically, workplace bullying may result in negative outcomes for employees, especially when this mistreatment threatens people’s resources (e.g., if an expectation of control is not met) (Hobfoll, 1989), or if the target believes the cause of the bullying resides within him/her (Weiner, 1986), indicating that internal locus of control orientation may not be as beneficial in bullying situations as one often expected under other circumstances and work stressors. Previous studies have shown that people with high internal locus of control experienced more negative consequences from stress when objective control was low (e.g., Kolb and Aiello, 1996). Given that on-going bullying has been described as a situation with reduced control for all targets (Zapf and Einarsen, 2005), we may expect it to particularly create negative outcomes among those with higher internal locus of control orientation, because their expectation of control is not met and because blaming others for one’s misfortune, hence holding an external locus of control orientation, may actually be more beneficial when facing bullying. People with high internal locus of control often engage in problem focused behaviors, like help-seeking and positive thinking, while those with high external locus of control more often engage in avoidance coping, like resignation (Gianakos, 2002). Although problem-focused strategies generally are assumed to be the best way to minimize stress (Hahn, 2000), it has been argued that it may not be so in work environments where stressors are outside the workers control (Pearlin and Schooler, 1978). In fact, emotion-focused strategies may be the best in reducing distress in situations like bullying, where the target has limited control over the situation (Fleming et al., 1984; Zapf and Gross, 2001). Illustrative of this, active coping styles and personal coping resources have turned out to be less beneficial when experiencing higher levels of bullying in a series of studies (Nielsen et al., 2008; Hewett et al., 2016; Reknes et al., 2016). In Hewett et al. (2016), as well as Reknes et al. (2016) studies, an active coping style was only beneficial at no or really low levels of bullying exposure. Individuals with high internal locus of control tend to use active coping strategies, which may make them more vulnerable, than those with high external locus of control, who use more passive strategies in low-control situations like workplace bullying (Hahn, 2000).

### Research Hypotheses

**FIGURE 1:**
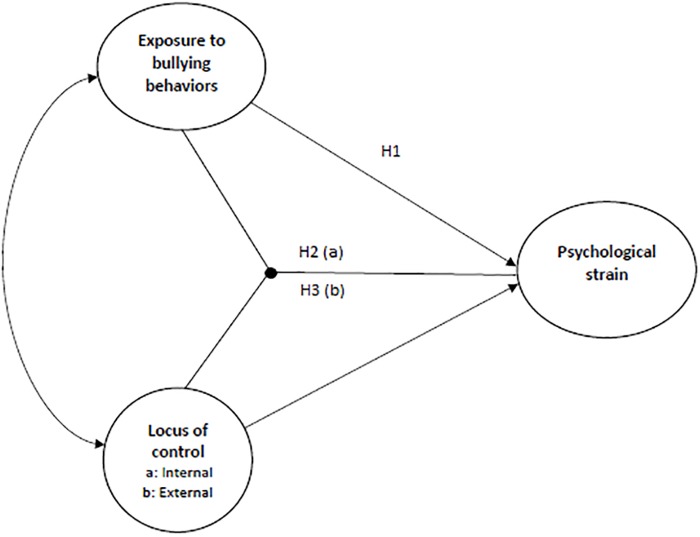
Theoretical model of internal and external locus of control, respectively, as moderators in the bullying-psychological strain relationship.

Even though theoretically it is reasonable to postulate that locus of control may act as an important moderator in the bullying-mental health relationship, this prediction has largely been ignored empirically (Rai and Agarwal, 2018). One exception is the study by Moreno-Jiménez et al. (2007), where external locus of control did not strengthen the hypothesized relationship between bullying and somatic and psychological health, indicating that this personal characteristic did not worsen the targets health when bullied. Other studies do also exist, however, using other yet similar constructs as the present study and with somewhat conflicting findings. For instance, Ariza-Montes et al. (2017) found that internal locus of control reduced job stress and strain among managers. Similarly, in Dijkstra et al. (2011), low internal locus of control strengthened the relationship between interpersonal conflict at work and psychological strain, indicating that high internal locus of control might act as a buffer variable. Also, in a study by Sassi et al. (2014) external locus of control interacted with quantitative workload on perceived stress, in the sense that high levels of external locus of control strengthened this relationship. In Schat and Kelloway (2000) study, however, locus of control did not moderate the relationship between workplace aggression and fear, nor did it moderate the relationship between fear and emotional well-being, somatic health, and neglect, respectively. Hence, even if some studies suggest that internal locus of control may act as a buffer in the work stressor-health relationship, while external locus of control strengthens this relationship, the results are somewhat inconsistent. Also, little research has been done on the role of locus of control in bullying situations so far, raising a need for more research studying these variables in conjunction (see Moreno-Jiménez et al., 2007 for an exception). Following this, the aim of the present study was to explain how and when exposure to bullying behaviors at work influences the health and well-being of those targeted, by looking at differences in perceived control over outcomes in life, using the concepts of internal- and external locus of control, respectively. To do so, three hypotheses were investigated building on the theoretical line of reasoning presented above (see Figure 1):

H1. Exposure to bullying behaviors is positively related to psychological strain.
H2. Internal locus of control acts as a catalyst in the relationship between exposure to bullying behaviors and psychological strain, in the sense that this relationship is stronger among targets holding an internal locus of control orientation.
H3. External locus of control acts as a buffer in the relationship between exposure to bullying behaviors and psychological strain, in the sense that this relationship is weaker among targets with an external locus of control orientation.

## Materials and Methods

### Design and Sample

A questionnaire survey fronted with information sheet was distributed electronically to Russian employees in 2014–2015 by internal Human Resource personnel within several organizations countrywide. All levels of the organizations were aimed for and regular biweekly reminders were sent to those invited to participate in the study. Participation was voluntary and actual participation seen as a statement of consent. The invitation to participate in the study was sent to 3365 employees. Altogether, 1474 responded (44% response rate), of whom 1048 (71.1%) were women and 426 (28.9%) were men. The mean age was 36.02 years (SD = 9.58). Furthermore, 83.7% had a higher education (Bachelor’s degree or higher), 14.8% had a technical degree, and 1.5% had finished middle or secondary school. In terms of organizational type, 85.9% worked in a private company, 13.7% worked in a local public organization, and 0.4% worked in a foreign public organization (see Table 1).

**Table 1:** A description of the study sample (*N* = 1474).

	*M*	SD	*n*	%
Age	36.02	9.58		
**Gender**				
Male			426	28.9
Female			1048	71.1
**Education**				
PhD			30	2.0
Master’s degree			537	36.4
Bachelor’s degree			667	45.3
Technical degree			218	14.8
Secondary school			17	1.2
Middle school			5	0.3
**Type of organization**				
Local private			1123	76.2
Local public			202	13.7
Foreign private			143	9.7
Foreign public			6	0.4

### Measures

Exposure to bullying behaviors was measured with the Negative Acts Questionnaire-Revised (NAQ-R; Einarsen et al., 2009). This scale consists of 22 items used to measure exposure to specific negative acts, with no reference to the phrase bullying (e.g., “Someone withholding information which affects your performance,” “Spreading of gossip and rumors about you”). The respondents were asked how often, during the last 6 months, they had been exposed to such negative behaviors at work. Responses were given on a 5-point scale from 1 (*Never*) to 5 (*About daily*). This scale showed a very strong internal consistency with a Cronbach’s alpha value of 0.96.

Psychological strain was measured with the 12-item version of the General Health Questionnaire (GHQ-12; Goldberg, 1978), measuring how the respondents had felt during the last 6 months (e.g., “felt capable of making decisions about things,” “been feeling unhappy or depressed”), on a response scale from 1 (*Not at all*) to 4 (*Much more than usual*). The Cronbach’s alpha value for this scale was 0.80, showing satisfactory internal consistency. GHQ-12 is evaluated to be a useful screening tool for the assessment of mental distress (Romppel et al., 2013).

Locus of control was measured with Levenson (1981) scale. Originally, this scale consists of three subscales; internal locus of control, powerful others, and chance. High scores on both the powerful others- and the chance subscales are thought to reflect an external locus of control orientation. In the present study, however, we applied the powerful others subscale as a measure of external locus of control. As the external- and the internal locus of control scale were uncorrelated in the present study (*r* = 0.05, *p* = n.s.), and a two-factor structure was supported in a confirmatory factor analysis (CFA; see below), these scales were used as independent measures of locus of control, as suggested by the scale author (Levenson, 1981).

Internal locus of control was measured with eight items (e.g., “whether or not I get to be a leader depends mostly on my ability”) and measures to what extent one feels in control over outcomes in life. Responses were given on a 6-point scale from 1 (*Strongly disagree*) to 6 (*Strongly agree*). The Cronbach’s alpha values for this subscale was 0.76, showing satisfactory internal consistency.

External locus of control (i.e., powerful others) was also measured with eight items (e.g., “I feel like what happens in my life is mostly determined by powerful people”), and assesses to what extent a person believes that outcomes in his/her own life are dependent upon powerful others. Responses were given on a 6-point scale from 1 (*Strongly disagree*) to 6 (*Strongly agree*), and the Cronbach’s alpha value for this subscale was 0.82, again showing satisfactory internal consistency.

### Statistical Analysis

The Statistical Package for Social Sciences (SPSS) 23.0. was used for analyzing demographics and scale reliability (α). Mplus 7.4. (Muthén and Muthén, 1998/2012) was used to perform confirmatory factor analysis and structural equation modeling (SEM). Fit indices used were root mean square error of approximation (RMSEA), Tucker-Lewis Index (TLI), and Comparative Fit Index (CFI). For CFI and TLI values above 0.95 indicate good fit, while values close to 0.08 for RMSEA indicate a satisfactory fit between measurement model and the observed data (Browne and Cudeck, 1993).

## Results

The construct validity of the included scales was investigated by means of a CFA in Mplus. The hypothesized measurement model (i.e., a 4-factor model with exposure to bullying behaviors, internal- and external locus of control, respectively, and psychological strain) was tested and compared with two alternative models. An inspection of the fit indices (Table 2) indicated that a five-factor model (see Table 3 for factor loadings), where the psychological strain scale was divided into two sub-scales (6 reversed positive items and 6 negative items), yielded the best fit (RMSEA = 0.05, CFI = 0.95, TLI = 0.95). However, the GHQ-12 scale has been argued to be a victim of the wording effect, and a unidimensional structure is preferred (Hankins, 2008; Ye, 2009). As such, we nested the two subscales into a second-order factor (i.e., psychological strain), to test how well this model fitted data. The results showed no deterioration in fit (RMSEA = 0.05, CFI = 0.95, TLI = 0.95). Hence, a four-factor model where psychological strain was treated as a second-order factor was used in further analyses.

**Table 2:** Fit statistics for confirmatory factor analysis (*N* = 1474).

Model	Latent factors	χ^2^	Df	CFI	TLI	RMSEA
4-factor model	WB, I_LoC, E_LoC, PS (one factor)	13911.67^∗^	1169	0.83	0.82	0.09
5-factor model	WB, I_LoC, E_LoC, PS (two sub-factors)	5023.82^∗^	1165	0.95	0.95	0.05
**4-factor model**	**WB, I_LoC, E_LoC, PS (second-order factor)**	**5080.51^∗^**	**1168**	**0.95**	**0.95**	**0.05**

*WB = workplace bullying, I_LoC = internal locus of control, E_LoC = external locus of control, PS = psychological strain. Measurement model is presented in bold.*

**Table 3:** Items and standardized factor loadings for the included variables.

Items	Factor loadings			
**Exposure to bullying behaviors**					
Withholding important information which affected your performance	0.76				
Humiliated or ridiculed in connection with your work	0.92				
Ordered to do work bellow your level of competence	0.73				
Had your key areas of responsibility and replaced them with more trivial or unpleasant tasks	0.79				
Had gossip or rumors spread about you	0.85				
Ignored or excluded	0.86				
Insulting or offensive remarks made about your person or attitudes, or private life	0.91				
Been shouted at or spontaneous anger and rage expressed at you	0.83				
Behaving in intimidating manner toward you, e.g., finger pointed at you, invaded personal space, shoved, blocked or barred your way	0.91				
Made hints and signals that you should quit your job	0.90				
Repeatedly reminded you of your errors or mistakes	0.81				
Ignored or faced you with hostile reaction when approached	0.92				
Persistently criticized your work	0.87				
Ignored your views and opinions	0.87				
Carried out practical jokes towards you by someone you don’t get on with	0.93				
Were given tasks with unreasonable or impossible targets or deadlines	0.75				
Had allegations made against you	0.87				
Had your work excessively monitored	0.73				
Someone pressured you not to claim what by right you were entitled to (sick leave, holidays, travel expenses)	0.87				
Someone made you a subject of excessive teasing or sarcasm	0.94				
You were exposed to unmanageable workload	0.74				
You had threats of violence or physical abuse, or actual abuse	0.98				
**Internal locus of control**					
Whether or not I get to be a leader depends mostly on my ability		0.47			
Whether or not I get into a car accident depends mostly on how good a driver I am		0.31			
When I make plans, I am almost certain to make them work		0.62			
How many friends I have depends on how nice I am		0.26			
I can pretty much determine what will happen in my life		0.73			
I am usually able to protect my personal interests		0.83			
When I get what I want, it’s usually because I worked hard for it		0.66			
My life is determined by my own actions		0.82			
**External locus of control**					
I feel like what happens in my life is mostly determined by powerful people			0.65		
Although I might have good ability, I will not be given leadership responsibility without appealing to those in positions of power			0.46		
My life is chiefly controlled by powerful others			0.80		
People like myself have very little chance of protecting our personal interests when they conflict with those of strong pressure groups			0.72		
Getting what I want requires pleasing those people above me			0.85		
If important people were to decide they didn’t like me, I probably wouldn’t make many friends			0.70		
Whether or not I get into a car accident depends mostly on the other driver			0.26		
In order to have my plans work, I make sure that they fit with the desires of people who have power over me			0.62		
**Psychological strain**					
Lost much sleep over worry?				0.67	
Felt constantly under strain?				0.72	
Felt you couldn’t overcome your difficulties?				0.69	
Been feeling unhappy or depressed?				0.89	
Been losing confidence in yourself?				0.90	
Been thinking of yourself as a worthless person?				0.87	
Been able to concentrate on what you are doing?					0.71
Felt you are playing a useful part in things?					0.71
Felt capable of making decisions about things?					0.79
Been able to enjoy your normal day-to-day activities?					0.89
Been able to face up to your problems?					0.83
Been feeling reasonably happy, all things considered?					0.61

**FIGURE 2:**
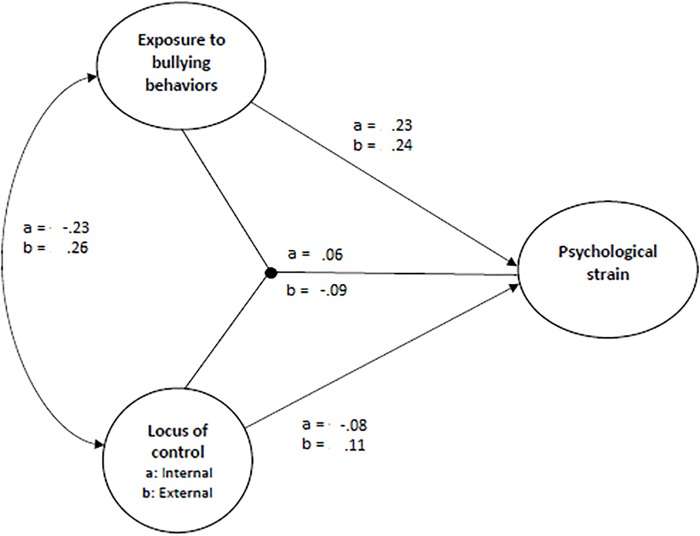
Results from the moderation analyses with latent factor interaction (standardized beta coefficients). a = results for internal locus of control. b = results for external locus of control.

**FIGURE 3:**
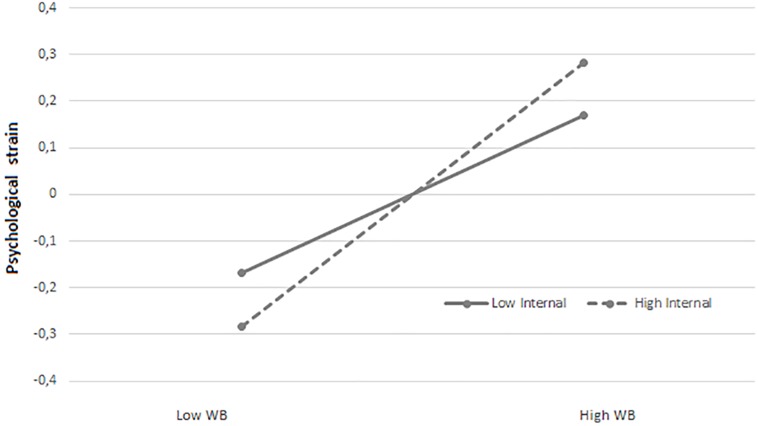
The interaction effect of workplace bullying and internal locus of control on psychological strain. Low = 1 SD below the mean. High = 1 SD above the mean. WB = workplace bullying. Internal = internal locus of control.

In order to test the relationship between locus of control, exposure to workplace bullying and psychological strain, SEM in Mplus was used. Firstly, the direct effect model was tested with bullying and internal locus of control as predictors of psychological strain (see Table 4). The fit indices indicated that the model had satisfactory fit to the data [χ^2^ (897, *N* = 1474) = 3493.39, *p* = 0.000; CFI = 0.96, TLI = 0.96, RMSEA = 0.04]. Then an interaction model was tested (see Figure 2, 3), with standardized variables, where bullying (β = 0.23, *p* = 0.000), internal locus of control (β = −0.08, *p* = 0.000), and the product term (Bullying^∗^Internal LoC: β = 0.06, *p* = 0.000) were related to psychological strain. The full model explained 7% of the variance in psychological strain. The relationship between bullying and strain was strongest for those with high scores on internal LoC (β = 0.28, *p* = 0.000) as compared with those having low scores (β = 0.17, *p* = 0.000). Hence, both H1 and H2 were supported.

**Table 4:** Fit statistics for the hypothesized relationships.

Model	Latent factors	χ^2^	Df	CFI	TLI	RMSEA	R^2^
Internal LoC							
Main model	WB, I_LoC, PS	3493.39^∗^	897	0.96	0.96	0.04	0.11
Interaction model	WB, I_LoC, WB^∗^I_LoC, PS						0.07
External LoC							
Main model	WB, E_LoC, PS	2584.20^∗^	897	0.98	0.97	0.04	0.12
Interaction model	WB, E_LoC, WB^∗^E_LoC, PS						0.08

*WB = Workplace bullying, I_LoC = Internal locus of control, E_LoC = External locus of control, PS = Psychological strain.*

**FIGURE 4:**
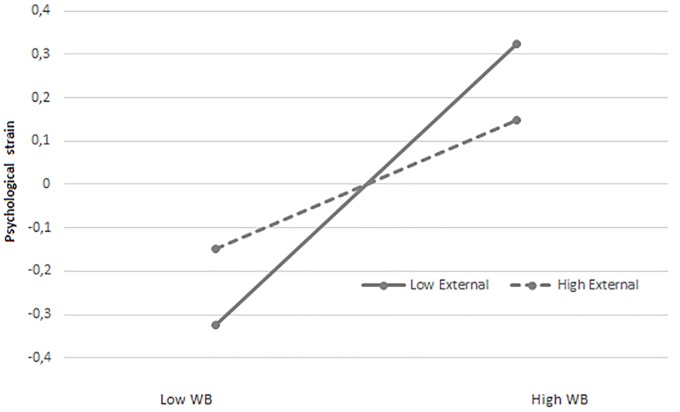
The interaction effect of workplace bullying and external locus of control on psychological strain. Low = 1 SD below the mean. High = 1 SD above the mean. WB = workplace bullying. External = external locus of control.

The third hypothesis, proposing that external locus of control acts as a buffer in the relationship between workplace bullying and psychological strain was also supported (see Table 4). The direct effect model with bullying and external locus of control as predictors of psychological strain yielded good fit to the data (χ^2^ (897, *N* = 1474) = 2584.20, *p* = 0.000; CFI = 0.98, TLI = 0.97, RMSEA = 0.04). In the interaction model, with standardized variables, both bullying (β = 0.24, *p* = 0.000), external locus of control (β = 0.11, *p* = 0.000), and the product term (Bullying^∗^External LoC: β = −0.09, *p* = 0.000) were related to psychological strain (see Figure 2, 4). The relationship between bullying and strain was strongest for those with low scores on external LoC (β = 0.32, *p* = 0.000) as compared with those having high scores (β = 0.15, *p* = 0.000). The full model explained 8% of the variance in psychological strain.

To sum up, the results in this study indicate that those with high external locus of control are less affected than those with low external locus of control, when exposed to bullying behaviors. Meanwhile, those with high internal locus of control are somewhat more affected than those with low internal locus of control, when exposed to higher levels of bullying behaviors at work. As such, our two main hypotheses are supported.

## Discussion

In the literature on workplace bullying, studies investigating when and for whom bullying results in negative outcomes are strongly called for (Nielsen and Einarsen, 2018; Rai and Agarwal, 2018). Hence, the aim of the present study was to explore the moderating role of internal and external locus of control, respectively, in the well-established bullying-mental health relationship. Based on theory and previous empirical research, we hypothesized that internal and external locus of control, respectively, may act differently than expected in this relationship, with high scores on external and/or low scores on internal locus of control acting as buffers that weaken the relationship between exposure to bullying and mental health. The assumption tested was that exposure to bullying is a situation where those targeted might have difficulties in altering or avoiding the on-going bullying (Zapf and Einarsen, 2005), hence being more difficult to handle for those with an internal locus of control and easier to live with for those high on external control. To investigate these postulations, SEM analyses in Mplus were conducted. The first hypothesis tested proposed that exposure to bullying behaviors was positively related to psychological strain (H1). This hypothesis was supported, in line with a range of studies around the globe over the last two decades (Hogh et al., 2011; Nielsen et al., 2014). Furthermore, internal locus of control was suggested to amplify the bullying-psychological strain relationship (H2), while external locus of control was suggested to act as a buffer in this relationship (H3). These latter hypotheses were both supported, as the relationship between bullying and strain was weaker among those with high external locus of control as compared to those with low external locus of control, and stronger among those with high internal locus of control compared to those with low internal locus of control. Yet, external locus of control seems to be the more important one in relation to strain, which is also supported by previous research (Gore et al., 2016).

### Locus of Control as a Moderator in the Stressor–Strain Relationship

The results in this study indicate that, when exposed to higher levels of bullying, the bullying-psychological strain relationship is strongest among those targets with high internal locus of control and among those with low external locus of control. This may be contrary to what one initially expects from the theory, given that generally internal locus of control is related to positive well-being outcomes, while external locus of control is related to negative outcomes (Sprung and Jex, 2012; Gore et al., 2016). One possible explanation for these results may be that bullying is perceived as something else and more severe than other demanding situations at work, for instance interpersonal conflicts and mere aggression (Reknes et al., 2017; Notelaers et al., 2018), in the sense that bullying is a situation where the target may not be able to alter nor avoid the on-going bullying (Zapf and Einarsen, 2005). Empirically, the results in the present study are in line with studies showing that people with high internal locus of control actually experience negative consequences from stress in situations of low objective control (e.g., Kolb and Aiello, 1996). As explained in the COR theory (Hobfoll, 1989), a loss of resources may cause strain. Hence, a sudden loss of control and rise in social pain typical for bullying situations may cause those with high internal locus of control to experience more strain than people with low internal locus of control, the latter generally experiencing less control over outcomes in their life. Moreover, Karasek (1979) theorized that work situation with high demands and low control was “a high stress job,” with unresolved strain levels. If bullying is a stressful situation for all, where individual coping mechanisms increasingly fail to work due to a gradually reduced control over the situation (see Zapf and Einarsen, 2005), one could argue that all targets would find the negative treatment equally stressful in the end. However, it may also be that those with high internal locus of control and low external control experience such a treatment as scarier and more threatening, because their loss of control is unexpected. Those low in internal control have lesser expectations to be in control over life’s events. Consequently, people with high internal locus of control in the end report almost as high distress level as those scoring low on internal locus of control, yet only under high exposure to bullying behaviors. Similarly, and in line with theory, we find that employees having an external locus of control report more psychological strain than those scoring low on this orientation under conditions of no or low bullying exposure. Yet, they seem to be relatively less affected when increasingly exposed to bullying behaviors. The finding that people high in external locus of control experience a lesser increase in strain when exposed to bullying behaviors compared to those low in external locus of control, may be explained by the tendency of those with high external locus of control to attribute outcomes on their lives to powerful others, more so than themselves. In general, this orientation is seen as disadvantageous, and has been related to stress and poor health (Evers et al., 2000; Gianakos, 2002), as well as negative well-being outcomes (Sprung and Jex, 2012) which is also seen in the present study. In more severe bullying situations, however, an external locus of control orientation, placing the cause of the bullying behaviors upon others (e.g., envy, bad manners or lack of self-control), may in fact be relatively advantageous as it protects one’s self-esteem for further deterioration and reduces any burden of self-blame. This result is somewhat in line with the study of Moreno-Jiménez et al. (2007), where external locus of control did not strengthen the relationship between bullying and somatic and psychological health outcomes, as hypothesized. Moreover, our findings show that those with low external locus of control ends up with the same distress level as those with high external locus of control when reporting higher exposure to bullying behaviors at work.

Another explanation for the findings in this study, may be due to the fact that people high in internal- and low in external locus of control tend to employ other coping styles when facing stressors, as those with high internal locus of control use problem-focused behaviors more often than those with high external locus of control (Gianakos, 2002). Normally, problem focused behaviors, are assumed to be the best way to minimize stress (Hahn, 2000), and one should anticipate that people high in internal locus of control would be better protected when using these strategies. However, in situations with limited control, like exposure to workplace bullying, emotion-focused strategies may be the best in reducing distress (Fleming et al., 1984; Zapf and Gross, 2001), because negative behavior is often outside the workers’ control (Pearlin and Schooler, 1978), occurring again and again over a long period of time. In fact, previous studies have shown that active problem solving coping styles and personal coping resources have turned out to be less beneficial when experiencing higher levels of bullying (Nielsen et al., 2008; Hewett et al., 2016; Reknes et al., 2016). Based on the present study, as well as previous studies, it seems that the effect of individual moderators in the bullying-mental health relationship are highly dependent on the intensity of the bullying and that high exposure to bullying is related to high levels of psychological strain for all targets irrespective of their personal resources. As such, it has been argued that workplace bullying is to be seen as a more traumatic experience than exposure to other stressors and leads to detrimental outcomes for all targets regardless of what personal resources they have available (Nielsen et al., 2016).

### Theoretical Implications

The present results have some important theoretical implications. First of all, the results challenge main stream theories which suggest that certain personality characteristics make people more vulnerable and other more resistant when facing stressors such as exposure to bullying. This does not seem to be the case, indicating either that such dispositions may act differently depending on the nature of the stressor or indicating that bullying is a stressor different from other typical social stressors at work. Hence, theoretical models should account for the fact that personal characteristics normally seen as negative/positive for people’s coping, health and well-being, may act differently than expected under exposure to bullying (see also Nielsen et al., 2008; Hewett et al., 2016). Theoretical models should also take into account that exposure to bullying, even in low doses, may be different from exposure to other demanding stressors at work (Notelaers et al., 2018). Future research should study if this is due to its proposed “no-control” nature or if there are other characteristics involved in bullying that may explain these findings.

### Practical Implications

The results in this study emphasize the importance of including moderators when studying the bullying-mental health relationship, in that the examination of direct effects may underestimate the impact of the predictor variable, at least its impact on some particular groups of targets. Also, even though certain personal characteristics are risk factors for poor health and well-being in general, they may in fact act in the opposite way when exposed to bullying behaviors. In particular, relying on individual resources that normally protect people from work stressors, in our case locus of control, seem not to reduce the risk of impaired health under exposure to bullying behaviors at work. For therapists, family physicians and counselors working with targets with health problems after bullying, this is important knowledge. Furthermore, organizational based anti-bullying policies and programs are often advised in studies like the present one, with proper policies and procedures to handle bullying complaints. Based on the present study, such programs are not to be implemented in order to protect some generally vulnerable workers, but should be developed as much for the protection of the otherwise healthy and stress resistant employees.

### Methodological Considerations

The use of cross-sectional data hinders the possibility to draw causal explanations for the findings in this study, and studies with longitudinal designs are recommended in order to conclude further. Although the use of self-report data is assumed to increases the risk of common method variance (Podsakoff et al., 2003), the findings in the present study are in line with those employing longitudinal designs (Reknes et al., 2016). Also, moderation models can be considered as casual by nature, even in cross-sectional studies, based on the underlying theories suggesting directional inferences which are intrinsically causal (Wu and Zumbo, 2008). Yet, the present study was carried out among Russian workers, with an overweight of female respondents (71%), which may limit the possibility to generalize the findings to other countries as well as to a pure male population. Moreover, the results may have been affected by the healthy worker effect (McMichael, 1976), as most of the respondents reported low levels of bullying exposure as well as low levels of psychological strain.

## Conclusion

Workplace bullying, even in less frequent forms, is related to reduced health and well-being among those targeted, possibly depriving its targets from experiencing control over outcomes in life. In line with such assumptions, the present study shows that this relationship is dependent upon the nature of the targets’ locus of control. Having an external locus of control seems to be beneficial when in this predicaments, as the relationship between bullying and strain were lower for these targets as compared to those low in external control. Targets with an internal locus of control, however, seem to fare worse when exposed to bullying. Those holding an internal locus of control orientation had a stronger relationship between exposure to bullying and strain, as compared to those low in internal locus of control. Hence, it seems that people high in internal- and people low in external locus of control are the most negative harmed when exposed for higher levels of workplace bullying. A possible explanation may be that when expected resources fail to work an increase in exposure to bullying behaviors results in greater harm than if the expectation of personal control is already absent. Having an external locus of control may also involve blaming others for one’s misfortune more than oneself, which may result in lesser feelings of shame and guilt. Hence, these targets report fewer symptoms of psychological strain when exposed to bullying behaviors.

## Ethics Statement

The study was approved by the ethics committee of the Department of Organizational Psychology at Birkbeck, University of London. Participation was voluntary and actual participation seen as a statement of consent.

## Author Contributions

All authors listed have made a substantial, direct and intellectual contribution to the work, and approved it for publication.

## Conflict of Interest Statement

SE holds training courses in the management of workplace bullying and advices employers on the prevention and management of workplace bullying. The remaining authors declare that the research was conducted in the absence of any commercial or financial relationships that could be construed as a potential conflict of interest.
